# Heparin and Insulin for Hypertriglyceridemia-Induced Pancreatitis: Case Report

**DOI:** 10.1100/tsw.2009.142

**Published:** 2009-11-01

**Authors:** Deepika Jain, John Zimmerschied

**Affiliations:** Department of Internal Medicine, University of Missouri, Columbia and Harry S. Truman VA Hospital, Columbia,Missouri, USA

**Keywords:** hypertriglyceridemia, acute pancreatitis, insulin, intravenous heparin

## Abstract

Hypertriglyceridemia is the etiology of acute nonbiliary pancreatitis in up to 3% of patients. Along with the supportive treatment of acute pancreatitis, treating the precipitating cause is important as well. There have been reports where heparin and insulin have been used for acute reduction of triglycerides, although there are no established guidelines for efficacy of these modalities. Heparin and insulin decrease triglycerides by stimulating lipoprotein lipase activity, which degrades triglycerides into fatty acids and glycerol. We present a case where a 54-year-old male presented with hypertriglyceridemia-induced acute pancreatitis. The serum triglyceride level was 10,320 mg/dl (normal: 0–15 mg/dl) at the time of admission. We started the patient on intravenous insulin and heparin infusion, and within 24 h of induction of treatment, the levels decreased by 50% to 5407 mg/dl. Thus, heparin and insulin can be considered a safe treatment modality for rapidly reducing triglyceride levels.

## INTRODUCTION

Hypertriglyceridemia-induced pancreatitis is a treatable condition. Apart from conventional methods of treatment, several less frequently used methods have been described in the literature to decrease the triglyceride levels rapidly, e.g., plasmapheresis, apo CII infusion, and use of insulin and heparin. Heparin and insulin are known to stimulate lipoprotein lipase activity, thereby reducing the levels of triglycerides. There are not many studies that establish the efficacy of these as conventional management, however. In our review of the literature, we found various reports of successful treatment of hypertriglyceridemia with insulin and heparin[1,2,3,4,5].

We report a 54-year-old male who was admitted with acute pancreatitis secondary to hypertriglyceridemia. Serum triglycerides at the time of admission were 10,320 mg/dl. We decided to start the patient on intravenous insulin and intravenous heparin. Within 24 h, the triglycerides decreased to 5407 mg/dl.

## CASE REPORT

A 54-year-old male presented to our hospital with acute epigastric pain. He had a past medical history of hypertriglyceridemia and diabetes mellitus. The patient reported progressively increasing epigastric pain for 1 day. The pain was severe and constant in character, and radiated to the back. It was associated with nausea and vomiting. The patient denied any fever or jaundice. He also denied any history of alcohol consumption, peptic ulcer disease, or cholelithiasis. The patient reported taking gemfibrozil (600 mg oral twice a day) at home.

The physical examination showed a temperature of 36.6°C, blood pressure of 130/87, respiratory rate of 16/min, and pulse rate of 75/min. The abdomen examination was significant for tenderness in the epigastric region. There was no palpable liver or spleen. Bowel sounds were present and normal. No evidence of an intra-abdominal mass or fluid was noted on physical examination. The examination of respiratory, cardiovascular, and nervous system was normal.

Relevant laboratory results at the time of admission were as follows. The serum was lipemic on gross examination. White blood cell count was 9300/cumm. Hemoglobin could not be measured due to lipemia. Serum triglyceride level was elevated at 10,320 mg/dl (0–15 mg/dl), serum cholesterol was 1061 mg/dl (0–200 mg/dl), and serum amylase and lipase were normal at 52 mg/dl (8–78 mg/dl) and 48 mg/dl (25–125 mg/dl), respectively. Blood glucose level was 331 mg/dl. Serum and urine ketones were negative. Serum anion gap was normal. Chest X-ray was normal. Abdominal X-ray was normal. USG of the abdomen was done, which showed no evidence of cholelithiasis. CT scan of the abdomen showed focal enlargement of the pancreas with mild peripancreatic inflammation. No fluid collection was seen. These findings were consistent with acute pancreatitis.

Conservative management was started and the patient was kept nil per mouth. We also started analgesics, antiemetics, and intravenous fluids. Following this, we started the patient on intravenous regular insulin infusion with 5% dextrose titrated for target blood sugars of <200 mg/dl and intravenous heparin infusion. The heparin was titrated for target PTT of <100 msec. The gemfibrozil and simvastatin were not continued as the patient was taking nothing by mouth.

The patient's abdominal pain and vomiting started to improve. Repeat serum triglycerides, 24 h later, had decreased to 5407 mg/dl and serum cholesterol level had decreased to 780 mg/dl.

During the hospital stay, his abdominal pain and triglyceride levels improved. He recovered with heparin and insulin infusion and did not require any other intervention. The heparin and insulin infusions were discontinued on day 3, once the patient reported clinical improvement and the triglyceride level had decreased to <2500 mg/dl. He was discharged from the hospital on day 6 with a triglyceride level of 1386 mg/dl. We could not normalize the triglyceride level at the time of discharge, but on follow-up, the triglyceride level reached 387 mg/dl 3 months postdischarge. He was discharged on simvastatin (80 mg oral every day at night) and fenofibrate (160 mg oral every day).

## DISCUSSION

The pancreas secretes digestive enzymes, amylase and lipase. When there is hypertriglyceridemia, the lipase acts on triglycerides present in the pancreas and converts them into free fatty acids. These fatty acids are nontoxic to the pancreas as long as they are bound to the albumin. However, when their levels increase such that albumin becomes saturated and free fatty acids are unbound in the pancreas, this leads to an inflammatory reaction and causes acute pancreatitis.

Lipoprotein lipase is an important enzyme that is needed for removal of triglycerides from the plasma. It is produced by endothelial cells of muscle and fat. The enzyme acts by degrading triglycerides into glycerol and free fatty acids, which accelerates the chylomicron degradation. Diabetes mellitus, alcohol abuse, or pregnancy can cause a secondary deficiency of lipoproteins, which can cause triglyceridemia and precipitate acute pancreatitis. The risk of hypertriglyceridemia-induced pancreatitis increases once serum levels are >1000 mg/dl[6].

At the same time, it is also important to recognize that triglyceride levels >500 mg/dl can falsely cause normal amylase levels, as it interferes with the calorimetric reading of the assay end point[7]. This was seen in our case as well, where the patient presented with acute pancreatitis (based on clinical features and findings on CT abdomen), but amylase and lipase were normal.

Henzen et al.[4] described successful treatment of hypertriglyceridemia-induced pancreatitis in five patients with heparin and insulin. They reported a mean decrease in serum triglyceride from a mean of 3822.2 to 888.8 mg/dl within a mean of 2.8 days.

Berger et al.[5] also reported five cases where serum triglyceride levels decreased to <500 mg/dl in less than 3 days in all five cases. Similarly, Jain et al.[1] reported two cases of successful treatment of hypertriglyceridemia with insulin and heparin.

In all the patients described in previous case reports, the mean decrease in triglyceride levels was about 50% within 24 h of use of intravenous insulin and heparin.

In our patient, serum triglyceride levels decreased by 50% within 24 h of starting heparin and insulin infusions. Our patient received insulin, heparin, and conventional antilipid medications (once he was able to tolerate oral intake) for treatment of hypertriglyceridemia-induced pancreatitis.

Thus, enhancing lipoprotein lipase activity by using heparin and insulin can help to reduce hypertriglyceridemia. These can be used as a safe and effective treatment modality, in conjunction with keeping the patient nil per mouth, in management of acute nonbiliary pancreatitis caused by hypertriglyceridemia. Even though nil per mouth can be sufficient to decrease the triglyceride levels in some instances, we need a more randomized evaluation in order to determine the nature of response to use of heparin and insulin in these patients.
